# ADAM9 enhances CDCP1 by inhibiting miR-1 through EGFR signaling activation in lung cancer metastasis

**DOI:** 10.18632/oncotarget.17648

**Published:** 2017-05-07

**Authors:** Kuo-Liang Chiu, Yu-Sen Lin, Ting-Ting Kuo, Chia-Chien Lo, Yu-Kai Huang, Hsien-Fang Chang, Eric Y. Chuang, Ching-Chan Lin, Wei-Chung Cheng, Yen-Nien Liu, Liang-Chuan Lai, Yuh-Pyng Sher

**Affiliations:** ^1^ Graduate Institute of Clinical Medical Science, China Medical University, Taichung 404, Taiwan; ^2^ Graduate Institute of BioMedical Sciences, China Medical University, Taichung 404, Taiwan; ^3^ Research Center for Tumor Medical Science, China Medical University, Taichung 404, Taiwan; ^4^ Division of Chest Medicine, Department of Internal Medicine, Taichung Tzu-Chi Hospital, Buddhist Tzu Chi Medical Foundation, Taichung 427, Taiwan; ^5^ School of Post-Baccalaureate Chinese Medicine, Tzu Chi University, Hualien 970, Taiwan; ^6^ Bioinformatics and Biostatistics Core, Center of Genomic Medicine, National Taiwan University, Taipei 100, Taiwan; ^7^ Center for Molecular Medicine, China Medical University Hospital, Taichung 404, Taiwan; ^8^ Division of Thoracic Surgery, China Medical University Hospital, Taichung 404, Taiwan; ^9^ Division of Hematology and Oncology, China Medical University Hospital, Taichung 404, Taiwan; ^10^ Graduate Institute of Cancer Biology and Drug Discovery, College of Medical Science and Technology, Taipei Medical University, Taipei 110, Taiwan; ^11^ Graduate Institute of Physiology, National Taiwan University, Taipei 106, Taiwan

**Keywords:** lung cancer, ADAM9, EGFR, miR-1, CDCP1

## Abstract

MicroRNAs (miRNAs), which are endogenous short noncoding RNAs, can regulate genes involved in important biological and pathological functions. Therefore, dysregulation of miRNAs plays a critical role in cancer progression. However, whether the aberrant expression of miRNAs is regulated by oncogenes remains unclear. We previously demonstrated that a disintegrin and metalloprotease domain 9 (ADAM9) promotes lung metastasis by enhancing the expression of a pro-migratory protein, CUB domain containing protein 1 (CDCP1). In this study, we found that this process occurred via miR-1 down-regulation. miR-1 expression was down-regulated in lung tumors, but increased in *ADAM9*-knockdown lung cancer cells, and was negatively correlated with *CDCP1* expression as well as the migration ability of lung cancer cells. Luciferase-based reporter assays showed that miR-1 directly bound to the 3′-untranslated region of *CDCP1* and inhibited its translation. Treatment with a miR-1 inhibitor restored *CDCP1* protein levels and enhanced tumor cell mobility. Overexpression of miR-1 decreased tumor metastases and increased the survival rate in mice. *ADAM9* knockdown reduced EGFR signaling and increased miR-1 expression. These results revealed that ADAM9 down-regulates miR-1 via activating EGFR signaling pathways, which in turn enhances *CDCP1* expression to promote lung cancer progression.

## INTRODUCTION

Lung cancer is the leading cause of cancer-related mortality [1]. Non–small cell lung carcinomas account for approximately 85% of lung cancers and have an overall 5-year survival of 15%, which is dependent in large part on the stage of disease at diagnosis [2]. In our understanding of tumor biology, dominant oncogenes exhibit interplay with tumor suppressor genes in pathogenesis and are involved in mediating tumor progression. These genes may offer new targets for biological therapies.

A disintegrin and metalloprotease 9 (ADAM9) and CUB-domain-containing protein 1 (CDCP1) are both oncogenic membrane proteins associated with lung cancer metastasis [3]. ADAM9, a type I transmembrane protein of the ADAM family, contains a disintegrin domain for adhesion and a metalloproteinase domain for ectodomain shedding [4–6]. Overexpression of ADAM9 in lung cancer cells is correlated with brain metastasis [7]. CDCP1, a cell surface glycoprotein for cell-cell interactions, promotes cancer metastasis and increase anchorage-free survival in lung adenocarcinoma [8]. Suppression of CDCP1 reduces tumor metastasis *in vivo*, demonstrating that blocking the function of CDCP1 influences tumor progression [8].

MicroRNAs (miRNA), which are short non-coding RNA molecules of 18–25 nucleotides, can modulate specific protein expression by targeting mRNA [9, 10]. Based on the rapid increase in the number of miRNAs identified, it is thought that more than one-third of human genes are regulated by miRNA [11]. Dysregulation of several miRNAs has been linked to the development of certain human diseases, including cancer. Aberrant expression of miRNAs, such as miR-1, has been detected in various types of clinical tumor specimens and cancer cell lines, and may be correlated with cancer development. Down-regulation of miR-1 has been detected in lung cancer [12] and hepatocellular carcinoma specimens [13] using methylation-mediated silencing of the miR-1 gene. Ectopic miR-1 expression reduced the tumorigenic properties and induced apoptosis of cancer cells, suggesting that miR-1 re-expression therapy is a potential strategy for suppressing oncogenes and arresting tumor development.

ADAM9 and CDCP1 are oncogenic membrane proteins that have been linked to cancer metastasis. Our previous study demonstrated that ADAM9 promotes lung cancer metastasis by enhancing the function of CDCP1 [3] and regulates the expression of several genes through dysregulation of miRNAs, such as activation of N-cadherin (*CDH2*; cadherin 2) and CDCP1 through miR-218 [14, 15]. In this study, we showed that ADAM9 enhances *CDCP1* expression by suppressing miR-1. Manipulating the dysregulated miRNA by targeting the ADAM9-CDCP1 axis can affect the progression of lung cancer.

## RESULTS

### ADAM9 suppresses miR-1 expression in lung cancer cells

**Figure 1:**
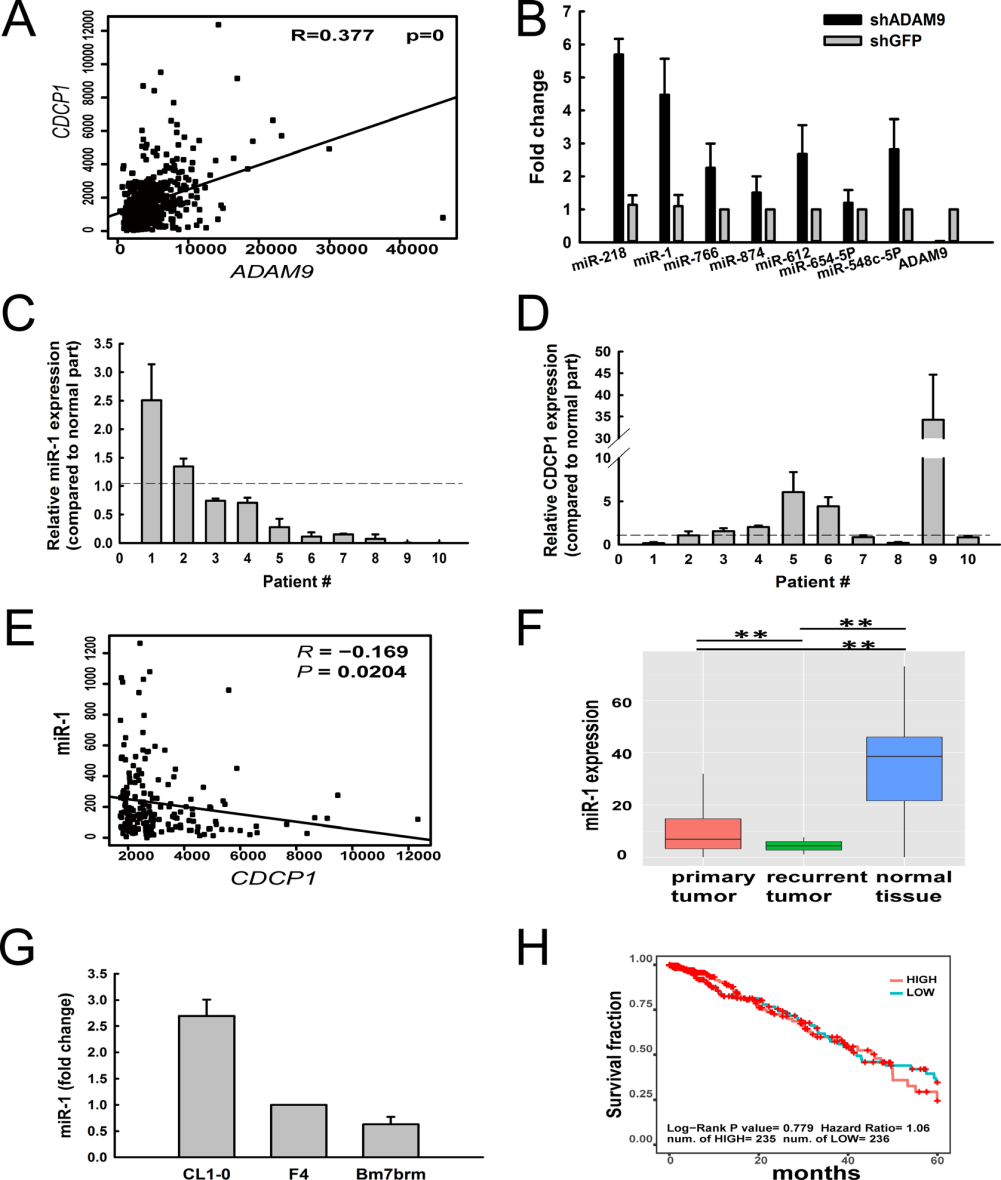
Expression of miR-1 was suppressed in lung tumor specimens, but increased in ADAM9-knockdown lung cancer cells (**A**) A positive correlation between *ADAM9* and *CDCP1* expression in lung cancer samples from the TCGA dataset (*n* = 519). (**B**) Relative expression levels of miRNA candidates predicted to target *CDCP1* in Bm7 lung cancer cells transfected with shRNA against *ADAM9* or GFP (control). GFP, green fluorescent protein. The expression levels were normalized to that of *U6B* RNA. Bars, SD. (**C** and **D**) Relative expression of miR-1 (C) and *CDCP1* (D) in lung cancer tissues from 10 patients, as compared to normal tissue counterparts. Bars, SD. (**E**) Negative correlation between miR-1 and *CDCP1* expression in lung cancer samples from the TCGA dataset (*n* = 519). (**F**) Box plot of miR-1 expression in primary lung tumor samples (*n* = 519), recurrent lung tumor samples (*n* = 2), and normal lung tissue samples (*n* = 46) from the TCGA dataset. **P < 0.01. (**G**) Quantitative RT-PCR analysis of miR-1 in lung cancer cell lines with increased migration ability. (**H**) Survival analysis of lung adenocarcinoma patients from the TCGA dataset, by miR-1 expression level.

In our previous study, we found that ADAM9 enhanced lung cancer migration by up-regulating CDCP1 and that blocking the two proteins reduced lung cancer metastasis [3]. Moreover, a significant positive correlation of *ADAM9* and *CDCP1* expression was detected in lung adenocarcinoma patients from The Cancer Genome Atlas (TCGA) dataset (*R* = 0.377, Figure 1A). To investigate whether miRNAs are involved in ADAM9's regulation of *CDCP*, we used miRNA microarrays to identify differentially expressed miRNAs in *ADAM9*-knockdown cells, and used the miRSystem [16] to predict miRNAs targeting *CDCP1*. Seven miRNA candidates were identified that had high expression in *ADAM9*-knockdown cells and were predicted to target *CDCP1*. All miRNAs and *ADAM9* RNA were validated using quantitative RT-PCR in control (shGFP) and *ADAM9*-knockdown cells (Figure 1B). The two miRNAs showing the highest fold-change were miR-218 and miR-1, and because miR-218 was assessed in our previous report [15], miR-1 was selected for further analysis in this study.

To determine whether miR-1 is dysregulated in lung cancer, we examined the endogenous expression levels of miR-1 in 10 primary clinical lung tumor specimens and found that most (80%) tumor samples exhibited lower expression levels of miR-1 than their normal counterparts (Figure 1C). In contrast, 50% of tumor samples showed higher levels of *CDCP1* in tumor cells compared to expression in normal cells from these samples (Figure 1D). Although miR-1 was not significantly negatively correlated with *CDCP1* in this small cohort, we observed a significant reverse correlation between miR-1 and *CDCP1* in lung adenocarcinoma from the TCGA dataset (Figure 1E). Furthermore, the level of miR-1 was highest in normal lung tissue and dramatically decreased in primary and recurrent lung tumors from the TCGA dataset (Figure 1F). Notably, miR-1 expression was significantly decreased in recurrent tumors compared to primary tumors, suggesting that this miRNA is involved in tumor progression. ADAM9 and CDCP1 were reported to show increased expression in cells with progressive migration ability (CL1-0 < F4 < Bm7brm) from the same original tumor [3]; we found that miR-1 expression was lower in the lung cancer cells with greater migration (Figure 1G). However, the level of miR-1 expression did not correlate with the overall survival of lung adenocarcinoma patients from the TCGA dataset (Figure 1H). Taken together, these results demonstrate that miR-1 expression is inhibited in lung cancer cells and can be restored in lung cancer cells by *ADAM9* knockdown.

### Suppression of ADAM9 decreases CDCP1 expression but increases miR-1 expression

**Figure 2:**
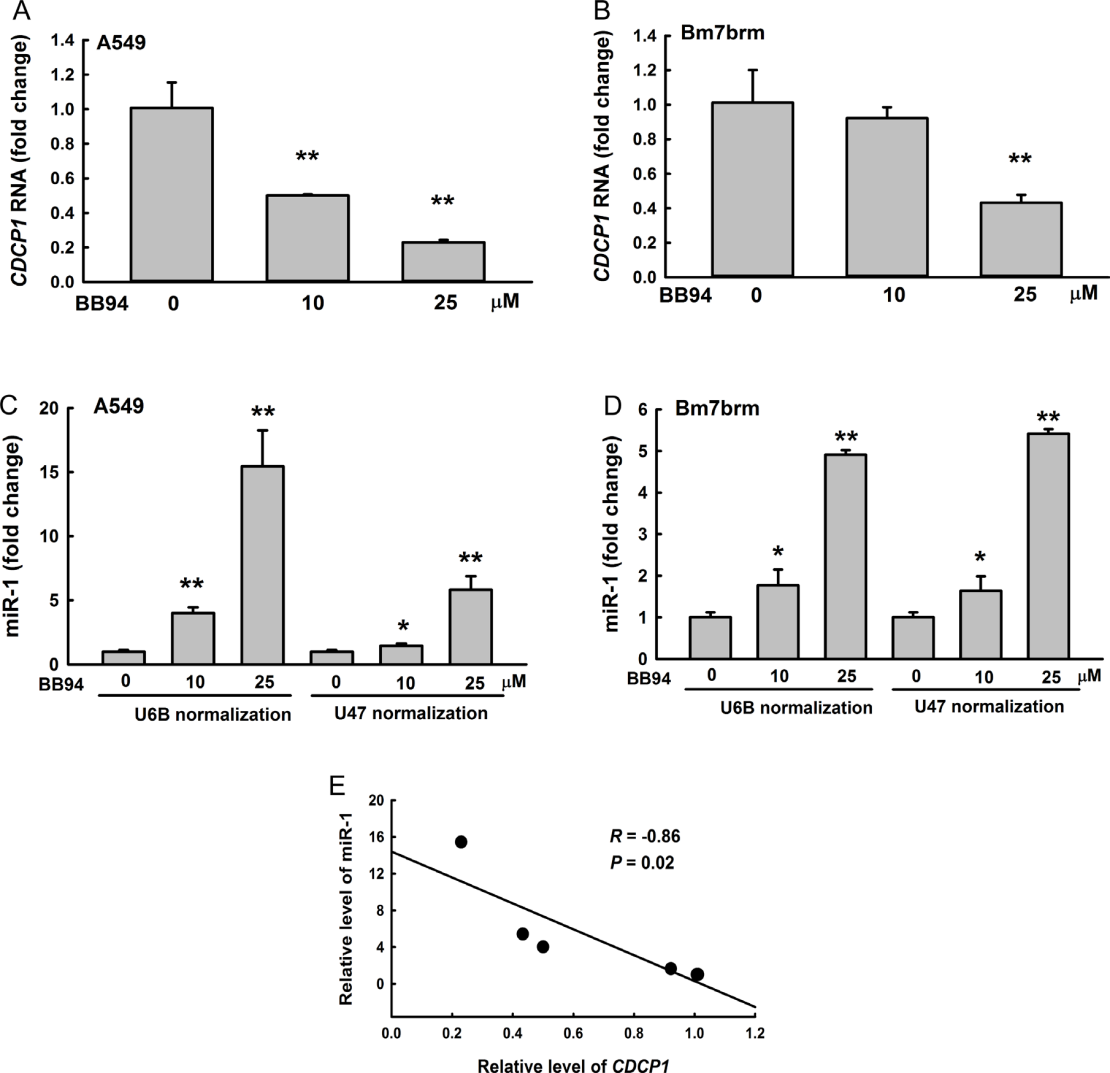
Negative correlation between *CDCP1* and miR-1 in lung cancer cells treated with BB94, a broad-spectrum inhibitor of metalloproteases (**A**, **B**) Quantitative RT-PCR analysis of *CDCP1* expression in A549 cells (A) or Bm7brm cells (B) treated with different doses of BB94. (**C**, **D**) Relative expression of miR-1 in A549 cells (C) or Bm7brm cells (D) treated with different doses of BB94 using *U6B* or *U47* small nuclear RNAs as a loading control. (**E**) Correlation between *CDCP1* and miR-1 expression in lung cancer cells treated with BB94. *P < 0.05. **P < 0.01.

ADAM9 proteins contain several major domains contributing to tumorigenesis, including a metalloproteinase domain. To explore whether metalloproteinase activity is important for miR-1 suppression in lung cancer cells, we treated the cells with the broad-spectrum metalloproteinase inhibitor BB94, which has been demonstrated to suppress ADAM9 expression [3], and then detected the expression levels of *CDCP1* and miR-1 by quantitative reverse transcription- PCR. *CDCP1* RNA expression was significantly decreased in A549 and Bm7brm cells treated with BB94 in a dose-dependent manner (Figure 2A and 2B). In contrast, miR-1 expression was significantly increased in lung cancer cells treated with BB94 (Figure 2C and 2D). Quantitative examination of BB94-dependent *CDCP1* RNA and miR-1 expression showed a negative correlation between *CDCP1* and miR-1 (correlation coefficient *R* = –0.86) (Figure 2E). Thus, the results indicated that ADAM9 can reduce miR-1 levels and increase *CDCP1* expression in lung cancer cells.

### miR-1 directly regulates CDCP1

**Figure 3:**
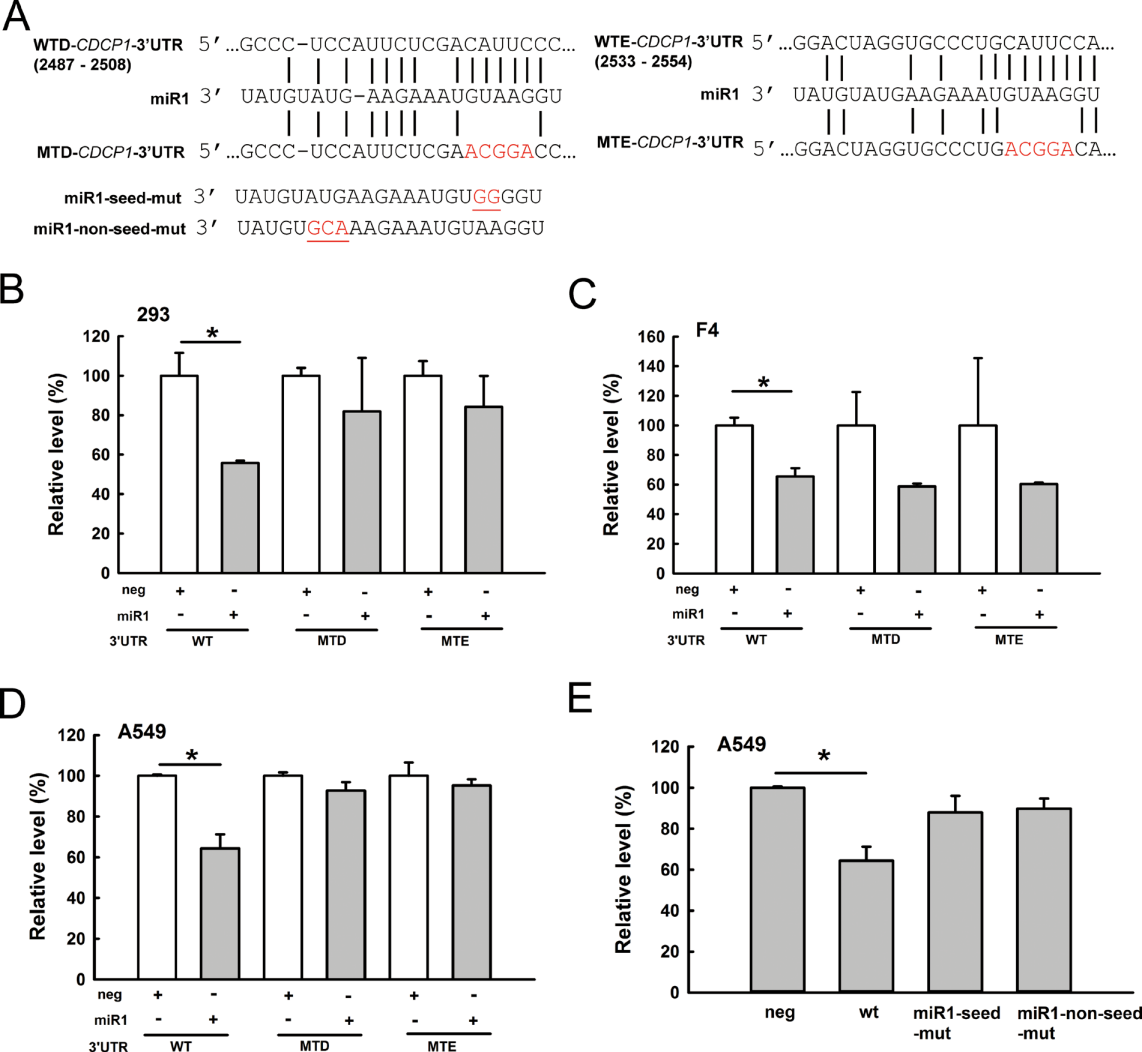
*CDCP1* is a target of miR-1 (**A**) Schematic representation of miR-1 targeting the *CDCP1* 3′-UTR. Two potential miR-1 binding sites located at 2487–2508 bp (site WTD) and 2533–2554 bp (site WTE) from the transcription start site of *CDCP1*. Mutated sequences at these two miR-1 binding sites are marked with red letters. miR-1 mutations at seed or non-seed sites are marked with red letters and underlines. (**B**–**D**) Luciferase assays of miR-1 binding to the wild-type and mutated *CDCP1* 3′-UTR in HEK 293 cells (B), F4 cells (C), and A549 cells (D). Cells were co-transfected with the plasmid of pri-mir-1, the firefly luciferase construct of *CDCP1* 3′-UTR, and *Renilla* luciferase control for the dual-luciferase assay. The relative luciferase activity represents the dual luciferase activity ratio (firefly/*Renilla* luciferase). WT: wild-type; MTD, MTE: mutation at sites sites D, E, respectively. (**E**) Luciferase assays of A549 cells co-transfected with pri-mir-1 wild-type and seed- or non-seed-mutants, the firefly luciferase construct of *CDCP1* 3′-UTR, and the *Renilla* luciferase control. *P < 0.05.

**Figure 6:**
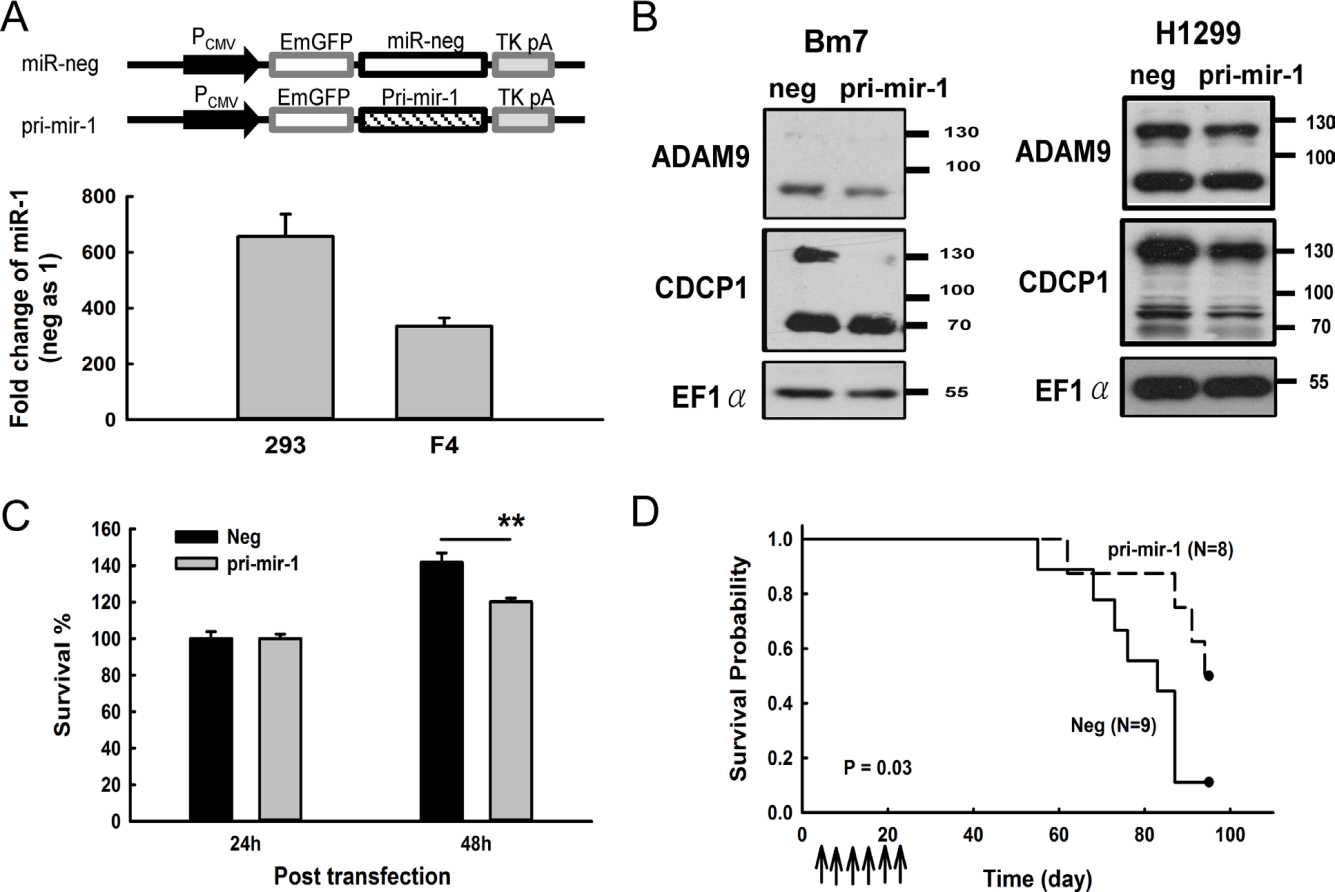
Overexpression of miR-1 decreased CDCP1 protein expression and prolonged survival time in mice bearing lung tumors (**A**) Ectopic expression of pri-mir-1 in HEK 293 and F4 cells. Schematic representation of the pri-mir-1 construct is shown at the top. The expression levels of miR-1 were detected 48 h after transfection and shown as the fold-change compared with the miR negative control. (**B**) Western blot analysis of CDCP1 in Bm7 and H1299 lung cancer cells transfected with plasmids of negative control and pri-mir-1. EF1α was used as an internal loading control. (**C**) Survival of Bm7 cells transfected with pri-mir-1 in anchorage-free culture. (**D**) Kaplan-Meier survival analysis of SCID mice bearing Bm7brmx2 tumors. Cancer cells (5 × 10^4^) were intracardially injected into mice and then tumor-bearing mice were treated with 25 mg of pri-mir-1-liposome complexes by intravenous injection twice per week for 3 weeks. Arrows indicate the time points of pri-mir-1-liposome therapy. **P < 0.01.

Because miR-1 was predicted to target *CDCP1*, we explored whether miR-1 can directly bind to the *CDCP1* 3′-UTR and inhibit *CDCP1*. Two potential miR-1 binding sites on the *CDCP1* 3′-UTR were predicted at 2487–2508 bp and 2533–2554 bp from the transcription start site. The *CDCP1* binding sites, and the seed region or non-seed region of miR-1, were mutated to determine the effects on translation (Figure 3A). We co-transfected the miR-1 plasmids (construct shown in Figure 6A) and different reporter constructs (as shown in Figure 3A) containing the *CDCP1* 3′-UTR following the luciferase gene into HEK 293 cells. The results showed that miR-1 inhibited luciferase activity compared to the empty vector control (neg) in HEK 293 cells (Figure 3B), F4 cells (Figure 3C), and A549 cells (Figure 3D). Furthermore, luciferase activity was not significantly reduced by miR-1 constructs with mutated binding sites (MTD and MTE; Figure 3B–3D) or by seed region or non-seed region mutants (Figure 3E). These results demonstrated that, regardless of the site, mutations in the *CDCP1* 3′-UTR or miR-1 relieved the suppression of translation. This suggests that miR-1 can directly bind to the 3′-UTR of *CDCP1* at two sites. Additionally, seed or non-seed sequences are critical for binding of miR-1 to the 3′-UTR of *CDCP1*.

### Manipulating CDCP1 protein expression by miR-1 affects cell migration

**Figure 4:**
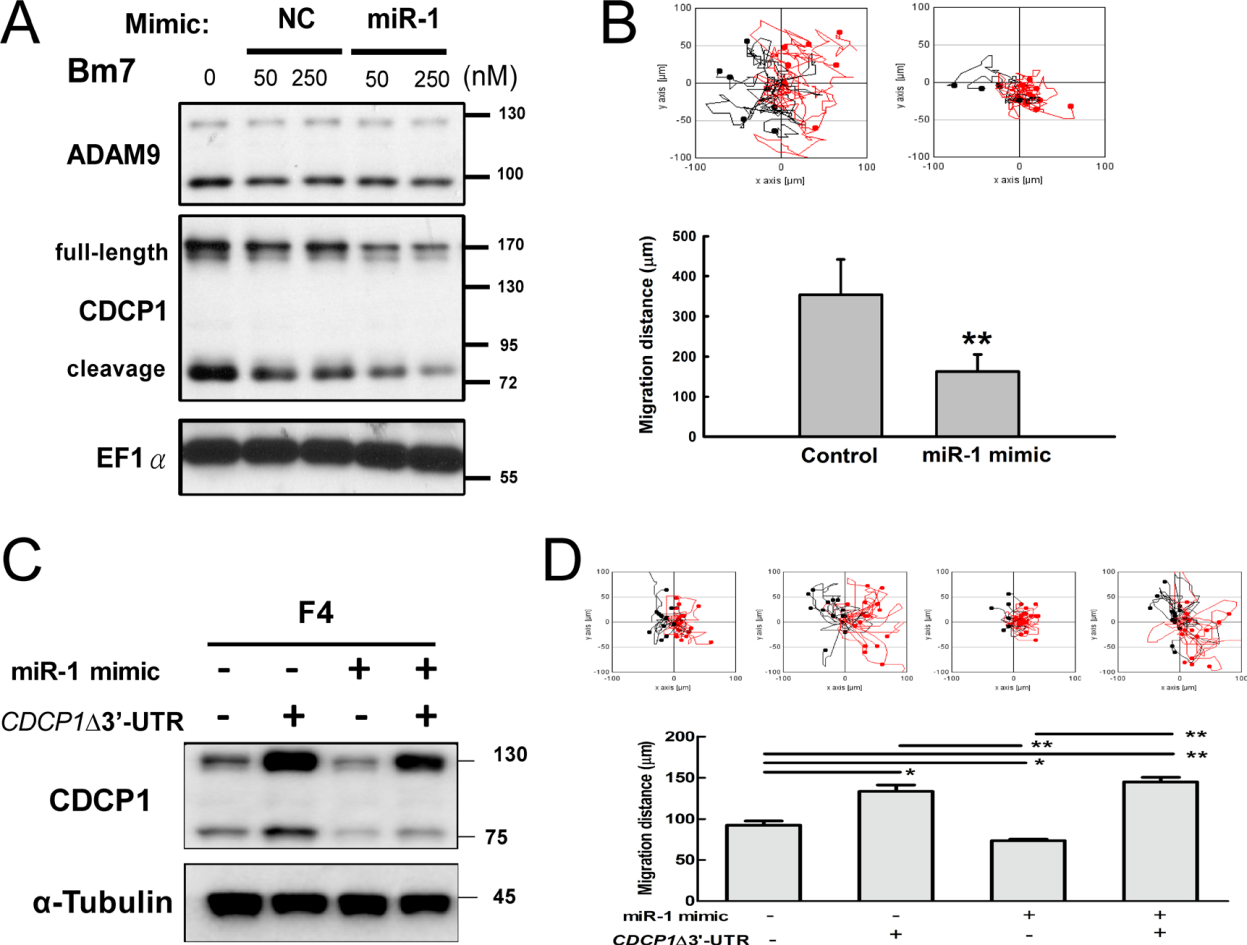
Ectopic expression of miR-1 decreased cell survival and migration ability in lung cancer cells (**A**) Western blot analysis of CDCP1 in Bm7 lung cancer cells treated with miR-1 mimic oligonucleotides. NC, negative control. Elongation factor 1 α (EF1α) was used as an internal control. (**B**) Migration ability of Bm7 cells transfected with miR-1 mimic (50 nM) using time-lapse video microscopy (top). Quantification of the 16-h migration distance (bottom). (**C**) Western blot analysis of CDCP1 in F4 cells overexpressing miR-1 mimic or/and plasmids of *CDCP1* lacking 3′-UTR (CDCP1D3′-UTR). (**D**) Migration ability of F4 cells transfected with miR-1 or/and plasmids of *CDCP1* lacking 3′-UTR using time-lapse video microscopy (top). Quantification of the 16-h migration distance (bottom). *P < 0.05. **P < 0.01.

To further examine whether miR-1 reduces CDCP1 protein expression via targeting the *CDCP1* 3′-UTR, and whether this inhibition influences cell migration, we transiently transfected a miR-1 mimic oligomers into Bm7 cells. CDCP1 protein levels were assessed by Western blotting, which allows detection of both the full-length CDCP1 (130 to 140 kDa) and its cleavage product (a 70-kDa C-terminal fragment [3]). The results showed that CDCP1 protein expression was decreased in miR-1 mimic-treated cells compared to negative control treatment (Figure 4A). In addition, the migration ability of lung cancer Bm7 cells transfected with the miR-1 mimic was significantly inhibited (Figure 4B). To further demonstrate that the reduction of cell migration by the miR-1 mimic was mediated by *CDCP1*, western blot analysis and cell migration assays were performed in F4 lung cancer cells overexpressing miR-1 mimic and/or *CDCP1* lacking the 3′-UTR (Figure 4C and 4D). High levels of CDCP1 were detected in F4 cells overexpressing *CDCP1* without 3′-UTR, but these were slightly reduced when cells were co-expressed with miR-1 mimic (Figure 4C). As expected, cell migration increased significantly along with increased ectopic expression of *CDCP1* without the 3′-UTR (Figure 4D, bar 2). Although cell migration was decreased by addition of the miR-1 mimic, the effect was lost in adding *CDCP1* without the 3′-UTR (Figure 4D, bar 4).

**Figure 5:**
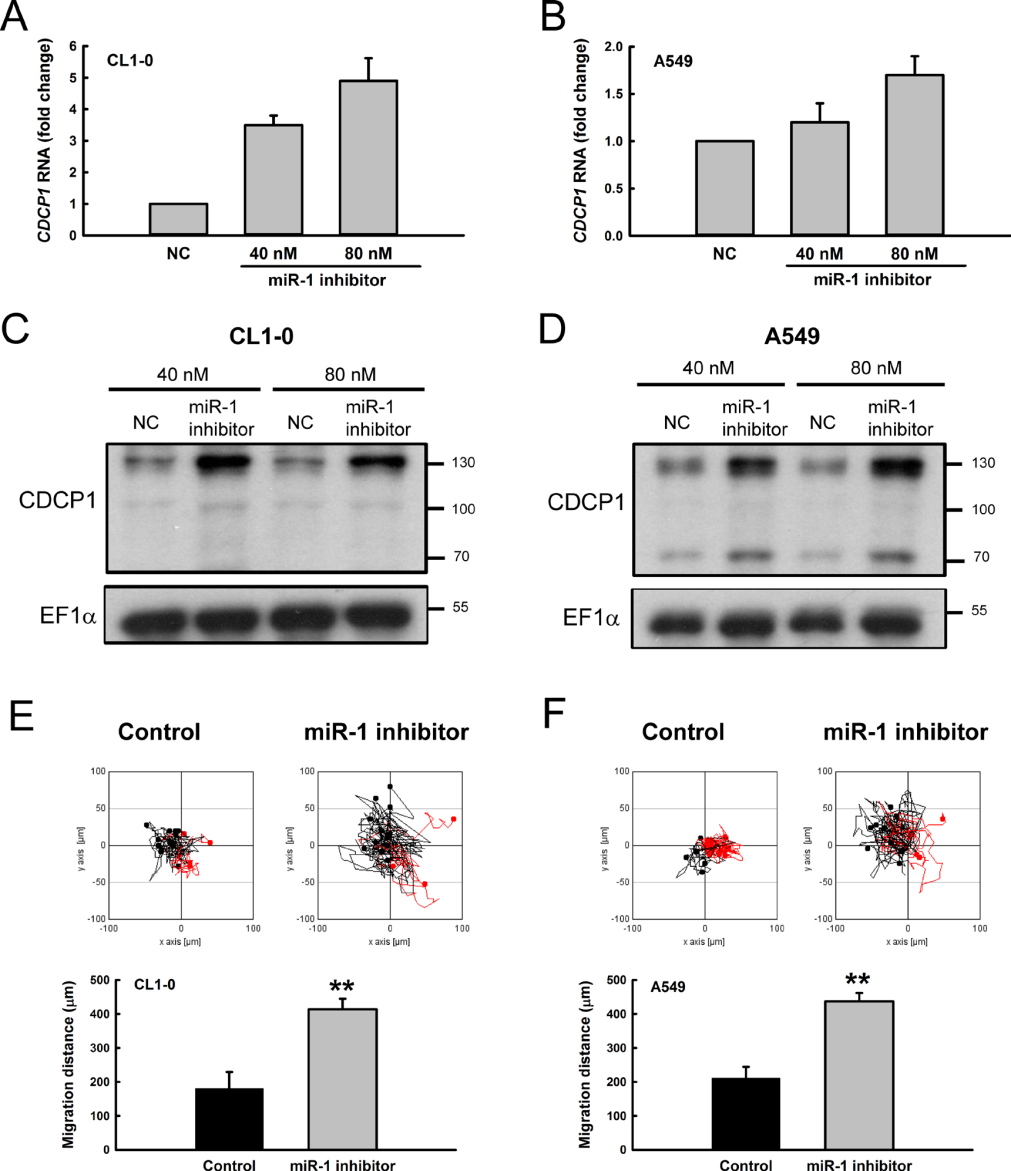
Treatment with miR-1 inhibitor increased CDCP1 expression and tumor cell mobility (**A**, **B**) Relative *CDCP1* RNA expression in CL1-0 cells (A) and A549 cells (B) with miR-1 inhibitor treatment. (**C**, **D**) Western blot analysis of CDCP1 expression in CL1-0 cells (C) and A549 cells (D) treated with miR-1 inhibitor. (**E**, **F**) Migration ability of CL1-0 cells (E) and A549 cells (F) transfected with miR-1 inhibitor (40 nM) using time-lapse video microscopy (top). Quantification of the 16-h migration distance (bottom). **P < 0.01.

When CL1-0 and A549 lung cancer cells were treated with a miR-1 inhibitor, both *CDCP1* RNA levels (Figure 5A and 5B) and CDCP1 protein levels (Figure 5C and 5D) were dramatically increased. In addition, the migration ability following miR-1 inhibitor treatment was significantly increased in CL1-0 cells (Figure 5E) and A549 cells (Figure 5F). These results indicate that miR-1 regulates CDCP1 protein expression and influences cell migration.

### Restoration of miR-1 inhibits tumor cell mobility and improves animal survival

To examine whether the restoration of miR-1 inhibits the tumorigenesis of cancer cells, we generated a construct by inserting the primary mir-1 (pri-mir-1) sequence behind the *EmGFP* gene sequence and found that the mature form of miR-1 was successfully processed when the construct was transfected into 293 and F4 cells (Figure 6A). A consistent pattern of decreased CDCP1 in pri-mir-1-transfected cells was observed in different lung cancer cell lines, such as Bm7, and H1299 (Figure 6B). In addition, cell survival was reduced in Bm7 lung cancer cells at 48 h after transfection with pri-mir-1 (Figure 6C). To further evaluate the antitumor effects of miR-1 *in vivo*, we established a metastatic lung tumor mouse model by intracardially inoculating with Bm7brmx2 lung cancer cells and then systemically delivering liposomal DNA complex containing pri-mir-1 twice per week for 3 weeks. The survival curve showed that pri-mir-1 plasmids significantly prolonged the survival time compared to the control group (Figure 6D). Thus, our results demonstrate that miR-1 has an antitumor effect in inhibiting the metastasis of lung tumors and prolonging animal survival time.

### ADAM9-mediated EGFR signaling reduces miR-1 expression

**Figure 7:**
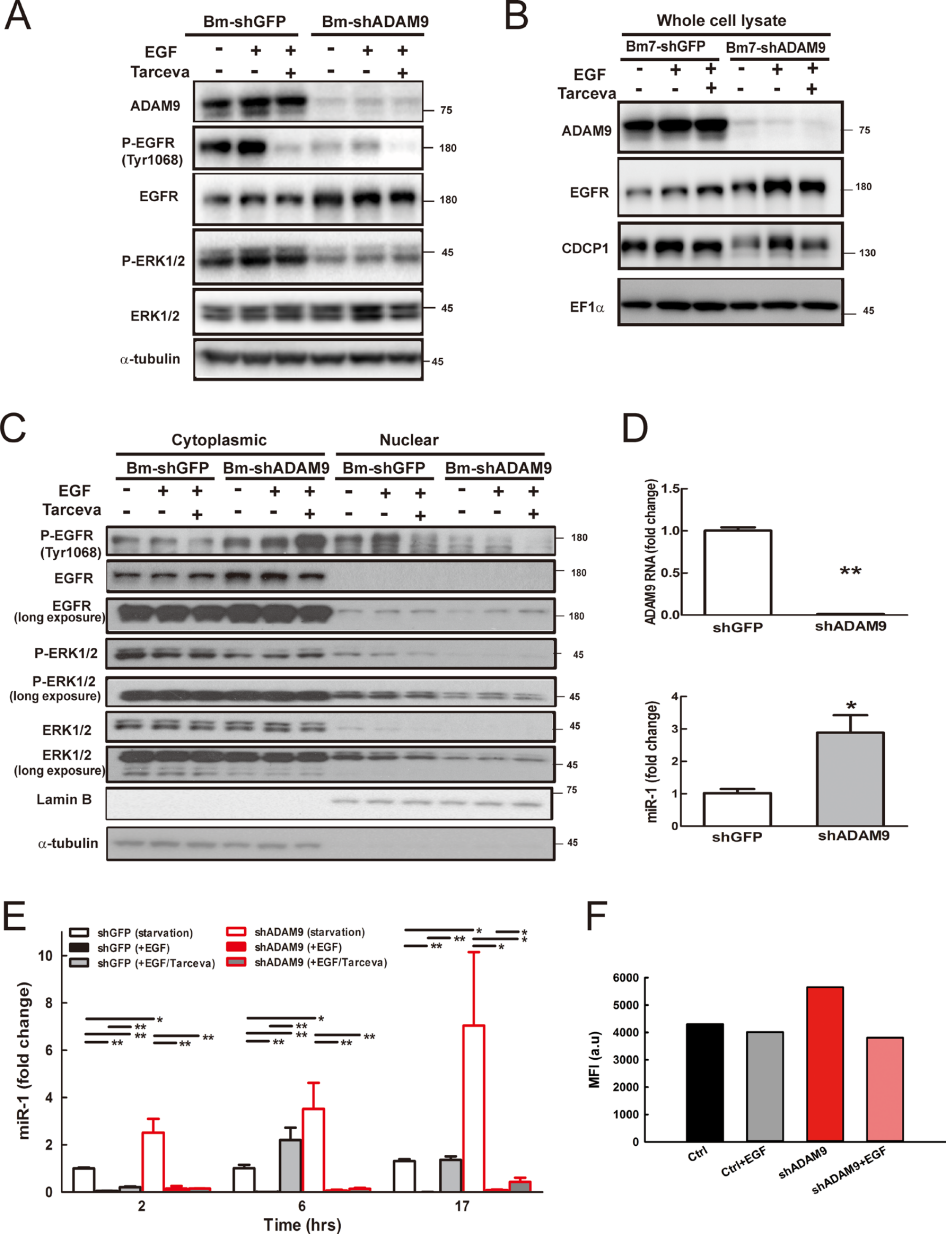
ADAM9 knockdown reduced EGFR signaling and increased miR-1 expression (**A**) Western blot analysis of ADAM9, EGFR, and ERK1/2 in whole cell lysate of control and ADAM9-knockdown cells treated for 6 h with EGF (100 ng/mL) or EGFR inhibitor Tarceva (15 μM). (**B**) Western blot analysis of ADAM9, EGFR, and CDCP1 in whole cell lysate of control and ADAM9-knockdown cells treated for 17 h with EGF (100 ng/mL) or EGFR inhibitor Tarceva (15 μM). EF1α, internal loading control. (**C**) Western blot analysis of phospho-EGFR (P-EGFR), EGFR, phospho-ERK1/2 (P-ERK1/2), and ERK1/2 in the cytoplasmic and nuclear fractions of control and ADAM9-knockdown Bm7 cells treated for 17 h with EGF or EGFR inhibitor Tarceva. EF1α, internal loading control; α-tubulin, cytoplasmic loading controls; Lamin B, nuclear loading control. (**D**) Relative *ADAM9* RNA and miR-1 expression in control and ADAM9-knockdown cells in serum-depleted culture. (**E**) Relative miR-1 expression in control and ADAM9-knockdown cells following EGF and EGF plus Tarceva treatment at different time points. (**F**) Promoter analyses of Bm7 cells transiently transfected with the pri-miR-1-2-RFP reporter and GFP plasmids (molar ratio 5:1) following EGF treatment. MFI, median fluorescence intensity. *P < 0.05. **P < 0.01.

EGFR signal activation is dependent on proligand shedding catalyzed by metalloproteases, such as ADAM, for production of soluble functional EGFR ligands [17]. Inhibition of ADAM family metalloproteases for modulating EGFR pathways may offer a potentially therapeutic strategy for human cancers [18]. Since the EGFR has been reported to promote prostate cancer metastasis to bone by down-regulating miR-1 [19], we further investigated whether ADAM9 may suppress miR-1 expression by activating EGFR signaling. First, we examined whether ADAM9 knockdown can reduce EGFR signaling. In serum starvation, the levels of phospho-EGFR and downstream phospho-ERK1/2, both indicators of EGFR signaling activity, were greatly reduced in ADAM9-knockdown cells (Bm7-shADAM9) compared to control cells (Bm7-shGFP) (Figure 7A, lane 4 versus 1). Phospho-EGFR and phospho-ERK1/2 were increased with EGF stimulation (lane 2 versus 1 and 5 versus 4) and the EGF-stimulated signals were reduced with EGFR inhibitor Tarceva treatment in Bm7-shGFP and Bm7-shADAM9 (lane 3 versus 2 and 6 versus 5); however, the signals were stronger in Bm7-shGFP than Bm7-shADAM9 (Figure 7A). In addition, CDCP1 expression was increased after EGF stimulation and decreased with Tarceva treatment (Figure 7B). Furthermore, we performed nuclear/cytosol fractionation to investigate whether nuclear EGFR can act as a transcriptional repressor to suppress miR-1 expression [19]. Increased nuclear phospho-EGFR translocation was observed upon EGF stimulation, but Tarceva inhibited the translocation in control Bm7-shGFP cells (Figure 7C). Similarly, in Bm7-shADAM9 cells, although the majority of phospho-EGFR remained in the cytoplasm after EGF stimulation, and Tarceva treatment reduced phospho-EGFR translocation, which resulted in higher phospho-EGFR detection in the cytosol. Reduced levels of phospho-ERK1/2 and total ERK1/2 were also detected in the nuclear fraction of ADAM9-knockdown cells. Thus, ADAM9 knockdown reduced EGFR phosphorylation and its nuclear translocation.

Next, we examined whether EGFR signaling regulates miR-1 expression over time in lung cancer cells treated with EGF or EGFR inhibitor. Under starvation and without any treatment, miR-1 expression was increased in ADAM9-knockdown cells at time 0 (Figure 7D) and miR-1 expression increased over time compared to control shGFP cells (Figure 7E, open bars). Upon EGF stimulation, miR-1 expression was significantly suppressed in both control and ADAM9-knockdown cells (unicolor filled bars). This effect was reversed with Tarceva after 6 h treatment in control cells. However, in ADAM9-knockdown cells, Tarceva treatment only partly rescued miR-1 expression after 17 h, and miR-1 remained at relatively low levels compared to control cells (Figure 7E, bicolor bars).

Two putative promoter regions upstream of the human primary miR-1-1 (pri-miR-1-1) and miR-1-2 (pri-miR-1-2) are known for encoding miR-1 transcripts [19]. From a promoter activity assay, we found moderately decreased pri-miR-1-2 promoter activity in ADAM9-knockdown Bm7 cells treated with EGF, and slightly decreased activity in control cells (Figure 7F). There was no difference in promoter activity of pri-miR-1-1 in cells treated with EGF (data not shown). Thus, EGFR signaling is likely to regulate the pri-miR-1-2 transcript in Bm7 cells.

**Figure 8:**
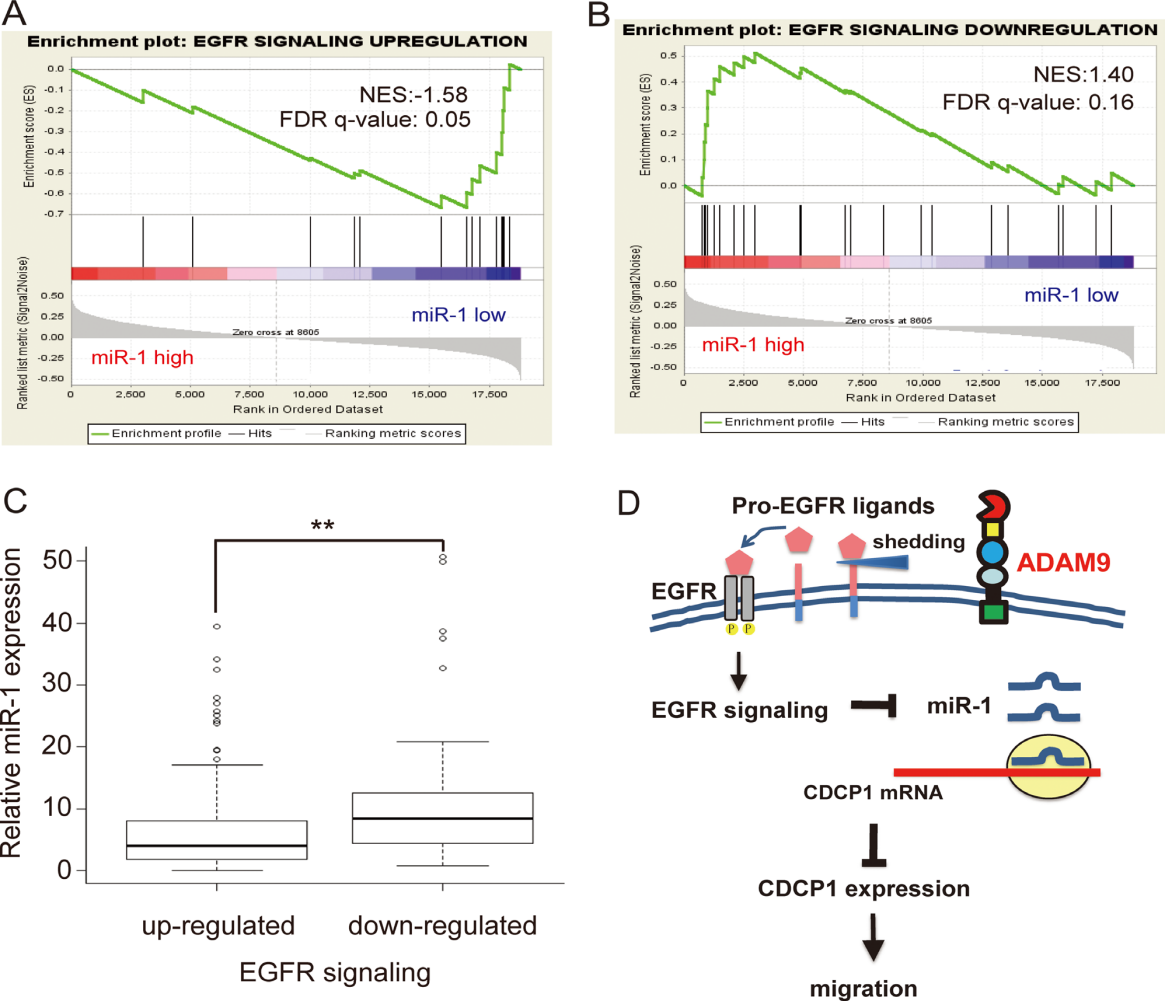
Negative association between EGFR signaling and miR-1 expression in TCGA lung adenocarcinoma (**A**) Gene Set Enrichment Analysis (GSEA) showed enrichment of the TCGA lung adenocarcinoma dataset with EGFR signaling up-regulation-responsive genes (*n* = 18) expressing lower levels of miR-1. NES, normalized enrichment score; FDR, false discovery rate. (**B**) GSEA showed enrichment of the TCGA lung adenocarcinoma dataset with EGFR signaling down-regulation-responsive genes (*n* = 25) expressing higher levels of miR-1 in cancer samples. (**C**) Relative expression of miR-1 in cancer versus normal tissues in the TCGA lung adenocarcinoma dataset, classified by up- or down-regulation of EGFR signaling gene signatures. **P < 0.01. (**D**) Model for ADAM9-enhanced CDCP1 expression by inhibition of miR-1 expression via activation of EGFR signaling.

In order to understand the correlation between miR-1 and EGFR signaling activity in clinical lung cancer specimens, we utilized the lung adenocarcinoma cancer data set containing both mRNA and miRNA data from TCGA and performed a gene set enrichment analysis (GSEA) [20]. In total, there were 505 lung adenocarcinoma samples and 19 normal samples. Among these 505 lung adenocarcinoma samples, cancer samples in which miR-1 expression was lower than the mean miR-1 expression of normal samples were defined as “miR-1 low” samples (*n* = 469); likewise, cancer samples in which miR-1 expression was higher than the mean miR-1 expression of normal samples were defined as “miR-1 high” samples (*n* = 36). The EGFR signaling up-regulation-responsive genes (*n* = 18) and down-regulation-responsive genes (*n* = 25) were collected based on published literature [21, 22]. The GSEA results showed that EGFR signaling up-regulation-responsive genes were correlated with “miR-1 low” cancer samples (Figure 8A). Conversely, EGFR signaling down-regulation-responsive genes were correlated with “miR-1 high” cancer samples (Figure 8B).

Furthermore, we examined the relative endogenous expression levels of miR-1 in cancer tissues with an up- or down-regulated EGFR signaling gene signature. The EGFR up-regulated group (*n* = 412) were selected by the criterion that the average expression level of EGFR signaling up-regulation-responsive genes in cancer samples was ≧1.5X greater than that of normal samples. Likewise, the EGFR down-regulated group (*n* = 229) was chosen based on whether the average expression level of EGFR signaling down-regulation-responsive genes in cancer samples was ≧1.5X lower than that of normal samples. After further excluding 176 cancer samples containing both up- and down-regulated EGFR signaling gene signatures, the endogenous level of miR-1 in each group was calculated. As shown in Figure 8C, the endogenous levels of miR-1 were significantly (*P* < 0.05) higher in samples with a down-regulated EGFR signaling gene signature. As shown in Figure 8D, our model suggests that ADAM9 promotes *CDCP1* expression by inhibiting miR-1 expression via activation of EGFR signaling. The stimulation of ectodomain shedding of pro-EGFR ligands by ADAM9 results in the activation of EGFR signaling, which represses miR-1 transcription and reduces its negative regulation of *CDCP1* expression. The resulting high levels of CDCP1 can promote the migration of lung cancer cells.

## DISCUSSION

Because the miRNA profile typically changes in lung cancer, it is important to determine whether oncogenic proteins, such as ADAM9, contribute to manipulating several critical miRNAs in cancer progression. Here, we demonstrated that miR-1 was regulated by ADAM9 and that miR-1 can directly bind to the 3′-UTR of *CDCP1*. Down-regulation of miR-1 led to *CDCP1* overexpression, promoting the malignancy of lung cancer cells. Restoring the miR-1 levels in lung cancer cells had an antitumor effect, as shown by decreasing cancer migration and metastasis. Our results reveal a novel regulatory mechanism of miR-1 involved in the ADAM9-CDCP1 axis in lung adenocarcinoma.

We found that ADAM9 also down-regulated several other miRNAs, such as miR-766 and miR-612, that target *CDCP1* in lung cancer cells. However, their roles in cancer progression may differ. For example, in hepatocellular carcinoma, miR-766 was down-regulated while miR-612 was up-regulated [23]. Up-regulation of miR-766 was correlated with favorable distant metastasis-free survival in triple-negative breast cancer patients [24]. In contrast, miR-766 has been reported to promote cell proliferation of human colorectal cancer by regulating *SOX6* expression [25]. The roles of these ADAM9-regulated miRNAs with potential to target *CDCP1* require additional investigation.

Although miR-1 is predominantly expressed in cardiac tissue and smooth and skeletal muscle [26], recent studies have revealed that miR-1 expression is often repressed in various types of cancer [27] and demonstrated therapeutic potential by targeting several oncogenes [28]. In lung cancer, ectopic expression of miR-1 reduced the protein levels of MET, Pim-1, FoxP1, and HDAC4 to influence the survival of cancer cells and oncogenic properties [12]. In addition, a negative correlation between miR-1 and *PIK3CA* expression was detected in nearly 70% of non-small cell lung cancer specimens. A combination of low miR-1 and high *PIK3CA* expression was highly linked to recurrence in patients after surgery [29]. Moreover, miR-1 represses genes such as *PNP* (purine nucleoside phosphorylase) and *PTMA* (prothymosin-α), leading to down-regulation of pathways regulating the cell cycle, mitosis, DNA replication, and actin dynamics in prostate cancer [30–32]. Notably, miR-1-mediated tumor suppressor effects are similar to the effect of histone deacetylase inhibitors.

DNA hypermethylation in the miR-1 gene silences miR-1 in colorectal cancers [33]. Using histone deacetylase inhibitors or ectopic expression of tumor suppressor C/EBPα (a member of the basic leucine zipper family of transcription factors) can re-activate miR-1 expression in lung cancer cells [12]. Therefore, dysregulation of miR-1 expression may be important for tumor development. In this study, we demonstrated that ADAM9 repressed miR-1. Inhibition of ADAM9 protein expression or protease activity rescued miR-1 suppression and down-regulated expression of its target *CDCP1* in lung cancer cells. Thus, miR-1 shows potential as a therapeutic agent for lung cancer.

ADAM9 promotes lung cancer metastasis through increase of *CDCP1* expression and activation of CDCP1 function [3]. Cell surface CDCP1 contributes to EGF/EGFR signaling-mediated migration in ovarian cancer with up-regulation of *CDCP1* RNA and protein expression [34]. Consistent with this, we found that EGF stimulation increases *CDCP1* expression and EGFR inhibitor reduces the level of CDCP1 in lung cancer cells. However, CDCP1 levels and EGFR signaling, as indicated by phospho-EGFR expression, were dampened in ADAM9-knockdown lung cancer cells, and ADAM9-knockdown cells showed more sensitivity to EGFR inhibitor treatment (Tarceva) than control cells (shGFP) (Figure 7A and 7B). Notably, the basal EGFR level was increased in ADAM9-knockdown cells, which might be a compensation for the loss of EGFR signaling. We found that miR-1 expression is increased in ADAM9-knockdown cells over time in serum free culture, but that enhancement was not found in control cells, which suggests other factors or pathways are also likely to regulate miR-1 expression in ADAM9-knockdown cells.

In our study, we found that ADAM9 dysregulates several miRNAs, such as miR-218 and miR-1, the top two miRNAs targeting the *CDCP1* 3′-UTR for reducing CDCP1 protein expression. From current studies, it is hard to tell which miRNA is most important in the regulation of CDCP1 by ADAM9 because they are expressed at similar levels in ADAM9-knockdown cells and have similar function to block cell migration.

In summary, we found that ADAM9 controls CDCP1 function by increasing its expression and activity, which results in lung cancer metastases. ADAM9 stimulates the plasminogen activator-based pathway to produce the cleaved form of CDCP1, which conducts stronger signaling to contribute to anchorage-free survival and cell metastasis [3]. In addition, we found that ADAM9 can increase CDCP1 expression through down-regulation of miRNAs.

## MATERIALS AND METHODS

### Cell culture and reagents

Human lung cancer cell lines (CL1-0, F4, Bm7, Bm7brmx2, A549, and H1299) were used in this study as previously described [14]. All cell lines were free of *Mycoplasma* contamination. The antibodies for western blotting, such as those against CDCP1 and ADAM9, were described previously [15].

### microRNA expression analysis and TCGA dataset analysis

The miRNA detection and analysis was performed as previously described [14]. Briefly, miRNA expression was determined using the Illumina human V2 miRNA expression bead chip (Illumina, San Diego, CA), and the data have been deposited in the Gene Expression Omnibus (GEO) database (accession number GSE51666).

Detailed information on the processing of the TCGA dataset is given in our previous studies [35, 36]. All samples of the TCGA lung adenocarcinoma (LUAD) dataset, including primary tumors (*n* = 519), recurrent tumors (*n* = 2) and adjacent normal tissues (*n* = 46), were selected for analysis in this study.

### Quantitative reverse transcription PCR of clinical specimens

*CDCP1* and *ADAM9* mRNA was quantified as previously described [15] and normalized against *GAPDH* (glyceraldehyde 3-phosphate dehydrogenase) mRNA. Lung tumor specimens were obtained from patients admitted to China Medical University Hospital (CMUH). Written informed consent was obtained in compliance with the protocols. All experiments were carried out in accordance with the guidelines approved by the CMUH Institutional Review Board.

### Plasmids, transfection, and generation of stable cell lines

The plasmid containing the *CDCP1* 3′-UTR was constructed as previously described [15]. The miR-1 binding sites were predicted in the *CDCP1* 3′-UTR using miRSystem [16]. Mutations in the miR-1 binding sites in the *CDCP1* 3′-UTR or in the primary miR-1 sequence were produced using the QuikChange Site-Directed Mutagenesis Kit (Agilent Technologies, Santa Clara, CA, USA) according to the manufacturer's protocol. The plasmids containing the *CDCP1* 3′-UTR, primary miRNA, and *Renilla* luciferase sequences were co-transfected into the indicated cells as previously described, and luciferase activity was measured using the Dual-Luciferase Reporter Assay System (Promega, Madison, WI, USA).

The primary sequence of miR-1, including the flanking precursor sequence (MI0000651), was amplified from human leukocyte DNA. The following primers containing *BamH*I and *Bgl*II restriction sites were used: 5′-CAGGGATCCTGTCCTGCTCACAC AGAGA-3′ (forward) and 5′-CCTAGATCTACAGG CAAAGTGACAGAACAATG-3′ (reverse). The nearly 400-bp PCR product was gel-purified and cloned into the *BamH*I-*Bgl*II sites of the pcDNA6.2-GW/EmGFP-miR-neg vector (Invitrogen, Carlsbad, CA, USA). The miR-1 mimic, miR-1 inhibitor, and negative control oligomers (Ambion, Austin, TX, USA) were transfected in cells as previously described [14].

### Time-lapse migration assay

This assay was conducted as previously described [3]. Briefly, cells were cultured on collagen-coated dishes (10 μg/mL, 3 mL) in serum-free media. Cell migration was captured using CCD video cameras (AxioCam MRm, Zeiss, Jena, Germany) at 20-min intervals for a total of 16 h with inverted microscopes (Axio Observer Z1, Zeiss). Accumulated migration distance was determined using the Track Point function of Image J software (NIH, Bethesda, MD, USA).

### Lung cancer animal model

The lung cancer animal model was established as previously described using protocols approved by the Institutional Animal Care and Use Committee of China Medical University and Hospital [15]. The therapeutic protocol, including intravenous injections of 100 μL of DNA:liposome complex containing 25 mg of plasmids, was similar to that used in a previous study [15].

### miR-1 promoter analysis and western blotting

The plasmids containing the promoter region of pri-miR-1-1 and pri-miR-1-2 were provided by Dr. Yen-Nien Liu, and this assay was conducted as previously described [19]. A plasmid mixture of promoter-red fluorescent protein (RFP) reporter and green fluorescent protein (GFP) (molar ratio 5:1) was transiently transfected into control and ADAM9-knockdown cells. After 16 h, cells were treated with EGF (100 ng/mL) for 6 h. GFP-expressing cells were gated to measure the MFI value for RFP by FACS.

In western blot analysis, nuclear and cytoplasmic fractions were separated and then used for protein detection as previously described [37].

### Statistical analysis

Statistical analysis was performed using Student's *t*-tests, and all statistical tests were two-sided. The Kaplan–Meier method was used for survival curves. The correlation coefficient was measured by Pearson Correlation Test. Statistical significance was set for all tests at *P* < 0.05.

## Abbreviations

ADAM9: a disintegrin and metalloprotease 9
CDCP1: CUB domain-containing protein-1
EGFR: epidermal growth factor receptor
miRNA: microRNA
TCGA: The Cancer Genome Atlas
UTR: untranslated region
